# Impact of cavernous internal carotid artery tortuosity on technical and clinical outcomes in mechanical thrombectomy for acute ischemic stroke: a single-center retrospective study

**DOI:** 10.3389/fneur.2026.1867423

**Published:** 2026-08-05

**Authors:** Zehra Uysal Kocabaş, Hicret Betül Adıyaman, Özlem Aykaç, Atilla Özcan Özdemir

**Affiliations:** 1 Department of Neurology, Faculty of Medicine, Eskisehir Osmangazi University, Eskisehir, Türkiye; 2 Department of Neurology, Sivas Numune Hospital, Ministry of Health, Sivas, Türkiye

**Keywords:** acute ischemic stroke, aspiration technique, cavernous ICA tortuosity, endovascular treatment, first-pass effect, mechanical thrombectomy

## Abstract

**Background and purpose:**

Cavernous internal carotid artery (ICA) tortuosity may hinder catheter navigation during mechanical thrombectomy (MT) for acute ischemic stroke (AIS). However, its impact on procedural and clinical outcomes remains unclear. This study evaluated the association between cavernous ICA tortuosity, classified by the Lin grading system, and outcomes in patients undergoing MT for middle cerebral artery (MCA) occlusion.

**Methods:**

In this single-center retrospective study, 425 patients with MCA occlusion treated with MT were included. Patients were categorized as tortuous (*n* = 205; Lin III–IV) or non-tortuous (*n* = 220; Lin I–II) based on lateral angiographic assessment. The primary outcome was successful recanalization within 60 min (puncture-to-recanalization). Secondary outcomes included first-pass success, final eTICI (2c–3), intracranial hemorrhage, new territory embolism, and functional outcomes (modified Rankin Scale [mRS]) at discharge and 90 days. Multivariate logistic regression identified predictors of first-pass success.

**Results:**

Tortuous anatomy was associated with lower first-pass success (43.9% vs. 54.1%; *p* = 0.036), fewer recanalizations within 60 min (47.3% vs. 57.3%; *p* = 0.040), and more procedural attempts (median 3 vs. 1; *p* = 0.025). Intracranial hemorrhage (35.6% vs. 20.5%; *p* < 0.001) and new territory embolism (5.4% vs. 1.8%; *p* = 0.048) were more frequent in the tortuous group. IA-tPA use was higher in tortuous patients (21.0% vs. 11.4%; *p* = 0.007). Good functional outcome (mRS 0–2) at discharge was significantly lower in the tortuous group (38.5% vs. 55.0%; *p* = 0.001). Cavernous tortuosity independently reduced first-pass success (OR = 0.643; 95%CI: 0.431–0.958; *p* = 0.030), while ASPECTS ≥6 (OR = 1.566; *p* = 0.040) and aspiration technique (OR = 3.682; *p* < 0.001) were positive predictors.

**Conclusion:**

Cavernous ICA tortuosity is an independent predictor of reduced first-pass success and worse early outcomes in MT. Aspiration may be a more effective strategy in this subgroup, supporting the importance of pre-procedural anatomical assessment.

## 1 Introduction

Mechanical thrombectomy (MT) has become the cornerstone of treatment for large vessel occlusion (LVO) acute ischemic stroke (AIS), with robust evidence demonstrating that timely recanalization within the first 24 h significantly improves neurological recovery by salvaging ischemic penumbra (1). While substantial progress has been made in refining patient selection, imaging protocols, and device technology, a critical yet underexplored determinant of procedural success remains vascular anatomy—particularly the morphology of the internal carotid artery (ICA) access route.

Previous studies have established that clinical factors such as age, baseline National Institutes of Health Stroke Scale (NIHSS) score, and serum glucose levels are important predictors of outcome following endovascular treatment (EVT) (2, 3). However, vascular factors—especially intracranial anatomical variations—have received comparatively less systematic attention, despite the fact that EVT is fundamentally a catheter-based intervention within the arterial lumen. The degree to which vessel tortuosity impacts procedural dynamics and clinical outcomes therefore warrants focused investigation.

The cavernous segment of the ICA, situated within the cavernous sinus and forming a characteristic S-shaped siphon with anterior and posterior genu connected by a horizontal segment, exhibits considerable inter-individual anatomical variability (4).

Tortuous configurations of the cavernous ICA may impede the advancement of guide catheters, intermediate catheters, microcatheters, and thrombectomy devices to their target positions, thereby delaying recanalization and potentially increasing the risk of procedural complications. Ribo et al. were among the first to formally document that difficult catheter access to the occluded vessel is associated with worse clinical outcomes in EVT (5).

Despite this early recognition, the literature on cavernous ICA tortuosity—as distinct from cervical ICA tortuosity—and its specific impact on MT outcomes remains sparse. A standardized classification system was proposed by Lin et al. (6), categorizing cavernous ICA morphology into four types based on the angulation and spatial orientation of the anterior and posterior genu, enabling reproducible grading from lateral angiographic projections. While this system has been applied in the context of intracranial aneurysm treatment via Pipeline embolization, its utility as a predictor of MT complexity in AIS has not been systematically examined.

Furthermore, the optimal thrombectomy technique in the setting of tortuous access anatomy—whether direct aspiration, stent retriever, or combined approach—remains an open question. Theoretical considerations suggest that aspiration may be less vulnerable to the geometric constraints imposed by sharp cavernous angulations, but empirical data are lacking.

The present study aimed to: (1) characterize the impact of cavernous ICA tortuosity (Lin classification types III–IV vs. I–II) on procedural efficiency, technical success, and clinical outcomes in patients undergoing MT for MCA occlusion; (2) identify independent predictors of first-pass recanalization success; and (3) evaluate the relative effectiveness of different thrombectomy techniques specifically within the tortuous cavernous ICA subgroup.

## 2 Materials and methods

### 2.1 Study design and patient population

This was a single-center, retrospective cohort study conducted at Eskişehir Osmangazi University Faculty of Medicine Hospital. We reviewed prospectively maintained records of consecutive patients who underwent MT for AIS between May 2015 and April 2025. The study was conducted in accordance with the Declaration of Helsinki; given the retrospective design and use of de-identified clinical data, a waiver of informed consent was granted by the institutional ethics committee.

Inclusion criteria were: age ≥18 years; symptom onset within 6 h; baseline NIHSS ≥5; premorbid modified Rankin Scale (mRS) 0–2; and MCA (M1 or M2 segment) occlusion confirmed on digital subtraction angiography (DSA) or CT angiography (7). Exclusion criteria included isolated ICA occlusion, tandem occlusions, posterior circulation occlusions, and incomplete procedural or imaging data.

Patients who were considered unsuitable for MT because of severe comorbidities, poor premorbid functional status, non-disabling symptoms, or other clinical contraindications were not included in the treated MT cohort. Therefore, the present analysis included only patients in whom MT was actually attempted and for whom complete procedural and imaging data were available.

### 2.2 Endovascular procedure

All procedures were performed by two experienced neurointerventionalists with at least 5 years of experience in endovascular stroke treatment, in a high-volume center performing approximately 300 mechanical thrombectomy procedures annually. Intravenous recombinant tissue plasminogen activator (rt-PA) was administered in eligible patients prior to thrombectomy. Thrombectomy technique—direct aspiration first-pass technique (ADAPT), stent retriever alone (SR), or a combined technique approach—was selected at the discretion of the treating operator. Intra-arterial tPA (IA-tPA) was used selectively as a rescue adjunct in cases of incomplete mechanical recanalization or distal embolization. The procedures were performed by neurointerventionalists with substantial experience in acute stroke intervention.

### 2.3 Cavernous ICA tortuosity classification

**Figure 1:**
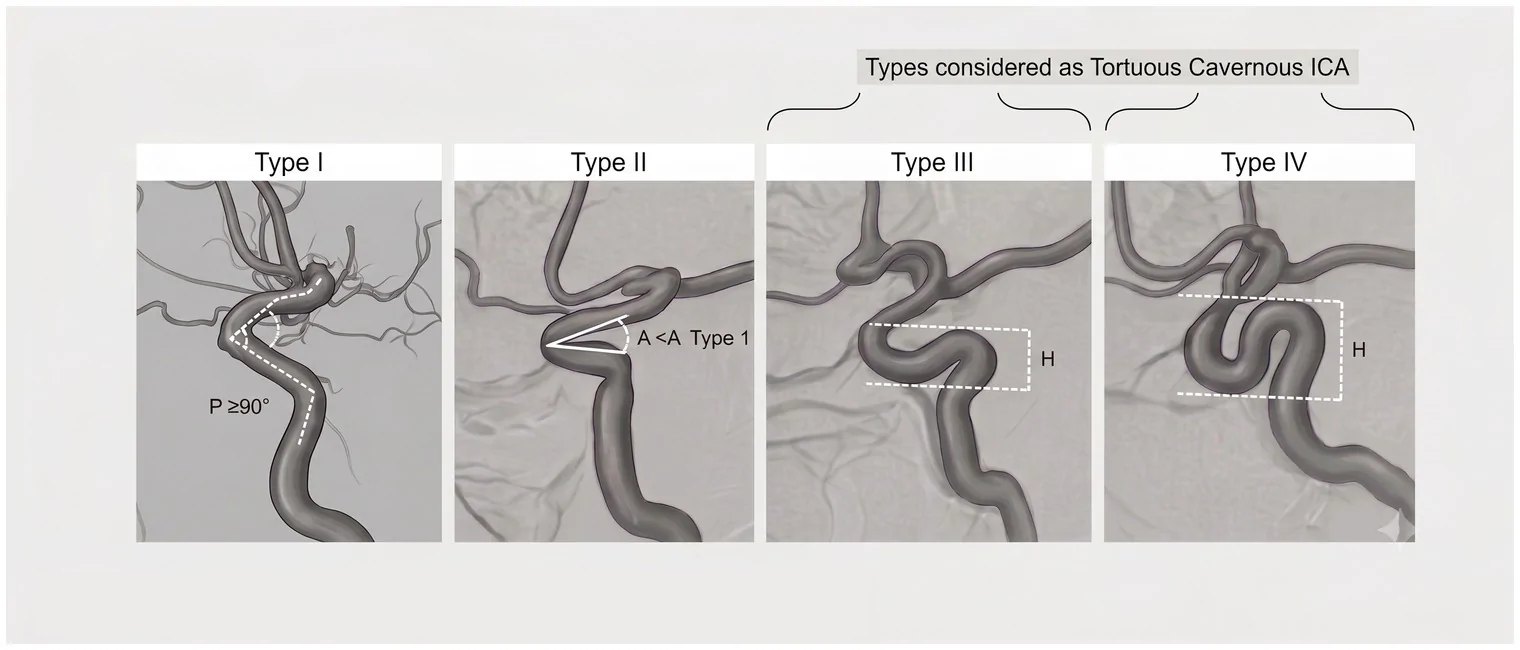
Schematic illustration of the classification of cavernous internal carotid artery (ICA) tortuosity.

Cavernous ICA morphology was graded on lateral DSA projections using the Lin classification system (6): Type 1, posterior genu angle ≥90°; Type 2, closed anterior genu with narrower angle compared to Type 1; Type 3, posterior genu oriented superiorly and posteriorly; Type 4, posterior genu positioned above the anterior genu. Types III and IV were designated as “tortuous” and Types I and II as “non-tortuous” for comparative analyses, consistent with the original publication’s designation of these configurations as predictors of procedural complexity. The tortuosity of the cavernous internal carotid artery was assessed by a single observer (6, 8) (Figure 1).

Cavernous ICA tortuosity was classified into 4 types based on the geometry of the anterior and posterior genus. Type I has open configurations/angles of anterior and posterior genus [the posterior genu angle (P) ≥ 90°]. Type II is characterized by a closed configuration of the anterior genu [more acute angle of the anterior genu (A) than type I]. Type III is defined by posterior deflection of the posterior genu, which gives it a buckled appearance. Type IV is the most tortuous and has a shape characteristic of the Simmons-style angiography catheter where the posterior genu is buckled superiorly compared with the anterior genu.^12^ H is the height difference of the anterior and posterior genus, measured from the peak of the posterior genu to the trough of the anterior genu. Types III and IV were considered tortuous cavernous ICA.

### 2.4 Outcome measures

The primary outcome was successful recanalization within 60 min of femoral puncture (puncture-to-recanalization time <60 min), chosen based on prior evidence demonstrating a significant increase in the probability of poor outcome beyond this threshold (9). Secondary outcomes included: first-pass recanalization rate (single-pass eTICI 2c–3); final eTICI score (successful recanalization defined as eTICI 2c–3) (10); number of procedural attempts; intracranial hemorrhage (ICH) classification per ECASS III criteria (11); new territory embolism; use of rescue IA-tPA; 90-day mortality; and functional outcome at discharge and 90 days, assessed by the mRS (good outcome: mRS 0–2).

Additional variables collected included: baseline NIHSS; ASPECTS on non-contrast CT (12); stroke etiology per TOAST classification (13); aortic arch type; bovine arch configuration; collateral status using the Tan angiographic score; imaging-to-puncture time; device characteristics [balloon guide catheter use, stent retriever diameter and length, distal aspiration catheter (DAC) size].

### 2.5 Statistical analysis

Descriptive statistics were reported as mean ± standard deviation (SD) for normally distributed continuous variables, median (range) for non-normally distributed variables, and counts with percentages for categorical variables. Between-group comparisons used the Mann–Whitney U test for continuous variables and Pearson’s chi-square or Fisher’s exact test for categorical variables, as appropriate. Differences in temporal parameters across thrombectomy technique subgroups in the tortuous cohort were assessed using the Kruskal-Wallis test. Binary logistic regression with variables selected *a priori* based on clinical relevance and univariate significance (*p* < 0.1) was used to identify independent predictors of first-pass recanalization success. Results are reported as odds ratios (OR) with 95% confidence intervals (CI). Statistical significance was set at *p* < 0.05. Analyses were performed using SPSS version 22.0 (IBM Corp., Chicago, IL, United States).

## 3 Results

### 3.1 Patient characteristics

**Figure 2:**
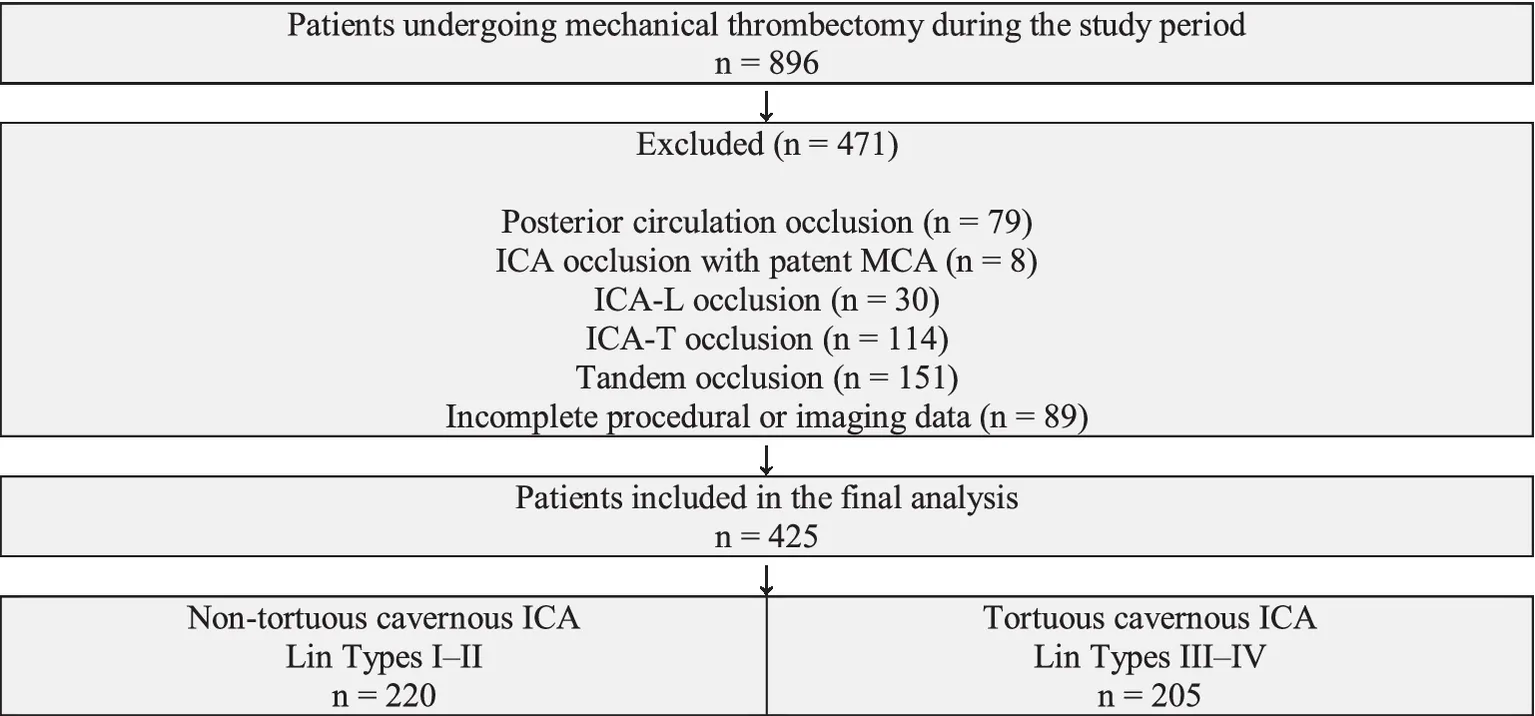
Study flowchart. Flow diagram illustrating patient selection, exclusion criteria, and final classification according to cavernous internal carotid artery tortuosity based on the Lin classification system. ICA, internal carotid artery; MCA, middle cerebral artery; MT, mechanical thrombectomy.

During the study period, 896 patients underwent mechanical thrombectomy. After exclusion of patients with non-eligible occlusion sites and those with incomplete procedural or imaging data, 425 patients with MCA occlusion and adequate angiographic visualization of the cavernous ICA were included in the final analysis (Figure 2).

A total of 425 patients were included: 220 in the non-tortuous group (Lin types I–II) and 205 in the tortuous group (Lin types III–IV). Baseline demographic characteristics were well-matched between groups. Mean age was 63.1 ± 12.9 years in the non-tortuous group and 63.7 ± 14.4 years in the tortuous group (*p* = 0.192). Male sex was present in 50.0% of non-tortuous and 52.2% of tortuous patients (*p* = 0.651). Baseline NIHSS was comparable (9 ± 7.9 vs. 9 ± 7.6; *p* = 0.789), as were rt-PA utilization rates (46.8% vs. 45.9%; *p* = 0.842), ASPECTS distribution (*p* = 0.219), collateral status (*p* = 0.143), and aortic arch morphology including bovine arch prevalence (10.9% vs. 9.3%; *p* = 0.575) (Table 1).

**Table 1:** Baseline demographic and imaging characteristics.

Variable	Non-tortuous (*n* = 220)	Tortuous (*n* = 205)	*p*-value	χ
Age (years), mean ± SD	63.1 ± 12.9	63.7 ± 14.4	0.318	21286.500
Male sex, *n* (%)	110 (50.0%)	107 (52.2%)	0.651	0.205
Baseline NIHSS, mean ± SD	9 ± 7.9	9 ± 7.6	0.789	22212.0
IV rt-PA use, *n* (%)	103 (46.8%)	94 (45.9%)	0.842	0.40
ASPECTS median (IQR)	5 (3–6)	4 (1–6)	0.197	20952.500
Good collaterals (≥50%), *n* (%)	145 (65.9%)	121 (59.0%)	0.143	2.148
Aortic arch type I, *n* (%)	90 (40.9%)	100 (48.8%)	0.191	3.312
Aortic arch type II, *n* (%)	122 (55.5%)	101 (49.3%)		
Aortic arch type III, *n* (%)	8 (3.6%)	4 (2.0%)		
Bovine arch, *n* (%)	24 (10.9%)	19 (9.3%)	0.575	0.314

### 3.2 Comorbidities and vascular risk factors

Several cardiovascular comorbidities were significantly more prevalent in the non-tortuous group compared to the tortuous group: obesity (23.2% vs. 11.2%; *p* = 0.001), prior coronary artery bypass grafting (CABG) (10.5% vs. 2.0%; *p* < 0.001), and coronary artery disease (CAD) (41.4% vs. 15.1%; *p* < 0.001). Conversely, a history of prior stroke was significantly more common in the tortuous group (22.9% vs. 9.5%; *p* < 0.001). Stroke etiology, atrial fibrillation, hypertension, diabetes mellitus, and smoking did not differ significantly between groups (Table 2).

**Table 2:** Cardiovascular risk factors and stroke etiology.

Variable	Non-tortuous (*n* = 220)	Tortuous (*n* = 205)	*p*-value	χ
Stroke etiology: atherosclerosis	165 (75.0%)	158 (77.1%)	0.112	4.377
Cardioembolic	43 (19.5%)	28 (13.7%)		
Undetermined	12 (5.5%)	19 (9.3%)		
Hypertension	122 (55.5%)	109 (53.2%)	0.637	0.223
Obesity	51 (23.2%)	23 (11.2%)	**0.001** *	10.560
Smoking history	16 (7.3%)	7 (3.4%)	0.079	1.803
Type 2 diabetes mellitus	63 (28.6%)	48 (23.4%)	0.221	1.499
Prior CABG	23 (10.5%)	4 (2.0%)	<0.001*	12.897
Coronary artery disease	91 (41.4%)	31 (15.1%)	<0.001*	35.706
Prior stroke	21 (9.5%)	47 (22.9%)	<0.001*	14.138

Bolded statistical significance is indicated as *p < 0.05.

### 3.3 Procedural and angiographic outcomes

First-pass recanalization was significantly more frequent in the non-tortuous group (54.1% vs. 43.9%; *p* = 0.036). The median number of procedural attempts was significantly higher in the tortuous group (median 3 vs. 1; *p* = 0.025). Successful recanalization within the first 60 min was achieved in 57.3% of non-tortuous patients compared to 47.3% of tortuous patients (*p* = 0.040). Although the overall rate of final successful recanalization (eTICI 2c–3) showed a numerical trend in favor of the non-tortuous group (68.2% vs. 61.5%), this difference did not reach statistical significance (*p* = 0.147). Puncture-to-microcatheter time and absolute puncture-to-recanalization time were numerically longer in the tortuous group but did not reach statistical significance (Table 3).

**Table 3:** Angiographic and procedural outcomes by tortuosity group.

Variable	Non-tortuous (*n* = 220)	Tortuous (*n* = 205)	*p*-value	χ
eTICI 2c–3, *n* (%)	150 (68.2%)	126 (61.5%)	0.147	2.104
First-pass recanalization, *n* (%)	119 (54.1%)	90 (43.9%)	0.036*	4.407
≥3 procedural attempts, *n* (%)	94 (42.7%)	104 (50.0%)	0.098	2.732
Recanalization within 60 min, *n* (%)	126 (57.3%)	97 (47.3%)	0.040*	4.217
Puncture-to-microcat. Time (min), mean ± SD	13 ± 16.8	13 ± 16.7	0.962	22492.0
Puncture-to-recanalization (min), mean ± SD	41 ± 19.6	46 ± 19.6	0.112	20537.50
Number of attempts, mean ± SD	1 ± 2.4	3 ± 2.6	0.025*	199.00

Bolded statistical significance is indicated as *p < 0.05.

### 3.4 Device and technique utilization

The rates of balloon guide catheter use, stent retriever use, stent retriever diameter, stent retriever length, and DAC size did not differ significantly between groups. IA-tPA use was significantly more frequent in the tortuous group (21.0% vs. 11.4%; *p* = 0.007). Among patients with tortuous cavernous ICA anatomy treated with primary aspiration and available catheter-size data, 6F aspiration catheters were used in 29 of 33 cases (87.9%), whereas 5F catheters were used in 4 cases (12.1%) (Table 4).

**Table 4:** Device and technique characteristics.

Variable	Non-tortuous (*n* = 220)	Tortuous (*n* = 205)	*p*-value	χ
Balloon guide catheter, *n* (%)	92 (41.8%)	97 (47.3%)	0.254	1.299
Stent retriever use, *n* (%)	170 (77.3%)	165 (80.5%)	0.418	0.657
Stent retrievers diameter ≤4 mm, *n* (%)	72 (32.7%)	71 (34.6%)	0.714	0.673
Stent retrievers length ≤25 mm, *n* (%)	51 (23.2%)	55 (26.8%)	0.723	1.327
DAC 6F, *n* (%)	132 (60.0%)	112 (54.6%)	0.506	0.305
IA-tPA use, *n* (%)	25 (11.4%)	43 (21.0%)	0.007*	7.295
Technique				
Combined	104 (47.3%)	92 (44.9%)	0.368	2.001
Contact aspiration	51 (23.2%)	40 (19.5%)		
Stent retriever alone	65 (29.5%)	73 (35.6%)		

DAC, distal aspiration catheter; IA-tPA, intra-arterial tissue plasminogen activator. Bolded statistical significance is indicated as *p* < 0.05.

### 3.5 Complications and functional outcomes

Intracranial hemorrhage occurred significantly more often in the tortuous group (35.6% vs. 20.5%; *p* < 0.001). Analysis of hemorrhage subtypes revealed a significant distribution difference (*p* = 0.009), with higher rates of petechial hemorrhage type 1 (10.7% vs. 4.5%), hematoma type 1 (8.3% vs. 4.5%), petechial hemorrhage type 2 (6.3% vs. 3.6%), and hematoma type 2 (5.9% vs. 2.7%) in the tortuous group. New territory embolism was significantly more frequent in the tortuous group (5.4% vs. 1.8%; *p* = 0.048). Vasospasm and dissection rates were comparable between groups. Good functional outcome (mRS 0–2) at discharge was significantly lower in the tortuous group (38.5% vs. 55.0%; *p* = 0.001). At 90 days, the difference in good functional outcome (60.0% vs. 66.8%) was no longer statistically significant (*p* = 0.145) (Table 5).

**Table 5:** Complications and functional outcomes.

Variable	Non-tortuous (*n* = 220)	Tortuous (*n* = 205)	*p*-value	χ
Any ICH, *n* (%)	45 (20.5%)	73 (35.6%)	<0.001*	12.153
Petechial hemorrhage type 1	10 (4.5%)	22 (10.7%)	0.009*	15.218
Hematoma type 1	10 (4.5%)	17 (8.3%)		
Petechial hemorrhage type 2	8 (3.6%)	13 (6.3%)		
Hematoma type 2	6 (2.7%)	12 (5.9%)		
SAH	11 (5.0%)	9 (4.4%)		
New territory embolism, *n* (%)	4 (1.8%)	11 (5.4%)	0.048*	0.446
Dissection, *n* (%)	8 (3.6%)	9 (4.4%)	0.692	0.157
Vasospasm, *n* (%)	74 (33.6%)	68 (33.2%)	0.919	0.010
mRS 0–2 at discharge, *n* (%)	121 (55.0%)	79 (38.5%)	0.001*	11.545
mRS 0–2 at 90 days, *n* (%)	147 (66.8%)	123 (60.0%)	0.145	2.129

Bolded statistical significance is indicated as *p < 0.05.

### 3.6 Multivariate predictors of first-pass success

Binary logistic regression analysis identified three independent predictors of first-pass recanalization success (Table 6). The presence of cavernous ICA tortuosity independently reduced the odds of first-pass success by 36% (OR = 0.643; 95%CI: 0.431–0.958; *p* = 0.030). ASPECTS ≥6 was associated with a 57% increase in odds of first-pass success (OR = 1.566; 95%CI: 1.022–2.402; *p* = 0.040). With combined technique as the reference, use of the ADAPT aspiration technique was associated with a 3.68-fold increase in first-pass success odds (OR = 3.682; 95%CI: 2.138–6.343; *p* < 0.001). Age, sex, and NIHSS were not independently associated with first-pass success (all *p* > 0.05).

**Table 6:** Multivariate logistic regression analysis—predictors of first-pass recanalization success.

Variable	*B*	SE	OR	95% CI	*P*-value
Cavernous ICA tortuosity	−0.442	0.204	0.643	0.431–0.958	0.030*
Age	−0.005	0.008	0.995	0.980–1.011	0.536
Female sex	−0.020	0.204	0.980	0.658–1.461	0.922
ASPECTS ≥6	0.449	0.218	1.566	1.022–2.402	0.040*
Baseline NIHSS	−0.002	0.013	0.998	0.973–1.024	0.898
ADAPT	1.303	0.277	3.682	2.138–6.343	<0.001*
Isolated stent retriever	0.291	0.233	1.338	0.847–2.113	0.212

Dependent variable: first-pass recanalization success (1 = yes, 0 = no). Reference for technique: combined approach. Bolded statistical significance is indicated as *p < 0.05.

### 3.7 Technique comparison within the tortuous subgroup

Among the 205 patients with tortuous cavernous ICA, three technique subgroups were compared: combined (*n* = 92), ADAPT aspiration (*n* = 40), and isolated stent retriever (*n* = 73). First-pass recanalization was significantly higher in the aspiration group (72.5%) compared to the combined (35.9%) and isolated stent retriever (38.4%) groups (*p* < 0.001). Similarly, recanalization within the first 60 min was most frequently achieved with aspiration (67.5% vs. 37.0% combined vs. 49.3% isolated SR; *p* = 0.005). Median puncture-to-recanalization time was significantly shorter with aspiration [41 (23–78) min] compared to combined technique [53 (17–95) min] and isolated SR [49 (15–90) min] (*p* = 0.004). Median procedural attempts were fewest with aspiration [median 2 (range 1–6)] versus isolated SR [4 (1–8)] and combined [4 (1–9)] (*p* < 0.001). Final eTICI ≥2c, hemorrhage rates, new territory embolism, vasospasm, and functional outcomes did not differ significantly across technique subgroups (Table 7).

**Table 7:** Clinical and angiographic outcomes by thrombectomy technique in tortuous cavernous ICA patients.

Variable	Combined (*n* = 92)	Aspiration (*n* = 40)	Isolated SR (*n* = 73)	*p*-value	χ
eTICI 2c–3, *n* (%)	52 (56.5%)	27 (67.5%)	47 (64.4%)	0.401	1.671
First-pass success, *n* (%)	33 (35.9%)	29 (72.5%)	28 (38.4%)	<0.001*	24.437
Recanalization ≤60 min, *n* (%)	34 (37.0%)	27 (67.5%)	36 (49.3%)	0.005*	19.099
Any ICH, *n* (%)	32 (34.8%)	13 (32.5%)	28 (38.4%)	0.804	2.293
New territory embolism, *n* (%)	53 (57.6%)	16 (40.0%)	42 (57.5%)	0.135	2.562
Vasospasm, *n* (%)	29 (31.5%)	10 (25.0%)	29 (39.7%)	0.255	4.297
mRS 0–2 at discharge, *n* (%)	33 (35.9%)	19 (47.5%)	27 (37.0%)	0.426	4.243
mRS 0–2 at 90 days, *n* (%)	53 (57.6%)	30 (75.0%)	40 (54.8%)	0.091	5.616
Puncture-to-recan. Time (min), median	53 (17–95)	41 (23–78)	49 (15–90)	0.004*	5302.5
Number of attempts, median	4 (1–9)	2 (1–6)	4 (1–8)	<0.001*	4270.50

Bolded statistical significance is indicated as *p < 0.05.

## 4 Discussion

This study presents a comprehensive analysis of the impact of cavernous ICA tortuosity on MT outcomes in a large, single-center cohort of 425 patients with MCA occlusion AIS. Our principal findings are fourfold. First, cavernous ICA tortuosity classified as Lin types III–IV independently reduces the probability of first-pass recanalization by approximately 36%, confirming its role as a meaningful anatomical determinant of procedural efficiency. Second, tortuous anatomy is associated with a significantly higher burden of procedural complexity—more device passes, less frequent recanalization within 60 min, and greater reliance on rescue IA-tPA. Third, procedural complexity translates into a significantly increased risk of intracranial hemorrhage and new territory embolism in the tortuous group. Fourth, and most importantly for clinical decision-making, the aspiration technique achieves markedly superior first-pass success rates in tortuous patients (72.5%) compared to combined or isolated stent retriever approaches, with a corresponding reduction in procedural time and number of passes.

The cavernous segment of the ICA is anatomically unique in its constrained location within the cavernous sinus and in the sharp angulations imposed by the anterior and posterior genu. The S-shaped siphon configuration inherently resists catheter advancement; when the posterior genu is rotated superiorly or the anterior genu is severely angulated (Lin types III–IV), the mechanical challenge is compounded. This is consistent with Ribo et al.’s seminal observation that difficult catheter access to the occluded vessel is associated with worse clinical outcomes (5), and with the broader literature on unfavorable vascular anatomy in EVT. Snelling et al. demonstrated that unfavorable vascular anatomy was associated with longer revascularization times and worse outcomes in anterior circulation thrombectomy (14), a finding corroborated by our data.

The first-pass effect (FPE)—defined as eTICI 2c–3 recanalization achieved in a single device pass—is increasingly recognized as the most important technical endpoint in MT, associated not only with better clinical outcomes but also with lower risks of embolization to new territories and fewer procedural complications (15, 16).

Our multivariate model confirms that cavernous ICA tortuosity is an independent negative predictor of FPE (OR = 0.643; *p* = 0.030), which aligns with findings from Koge et al., who demonstrated in a prospective multicenter study that ICA tortuosity significantly impacted MT procedural metrics (8). The association between ASPECTS ≥ 6 and first-pass success should be interpreted as a statistical association rather than as a direct causal effect; higher ASPECTS may reflect more favorable baseline tissue status and thrombus-access conditions that indirectly accompany successful reperfusion.

Our study extends this work specifically to the cavernous ICA segment, using a validated morphological classification, and in a substantially larger cohort.

The significantly higher rate of new territory embolism in the tortuous group (5.4% vs. 1.8%; *p* = 0.048) is a mechanistically coherent finding. In tortuous anatomy, the increased friction between the catheter/device and vessel wall, combined with the difficulty of maintaining co-axial alignment of the thrombectomy system, likely increases the probability of clot fragmentation during retrieval. Kim et al. demonstrated in a 3D-printed *in vitro* model that vascular tortuosity substantially altered the biomechanics of thrombectomy devices, with stent retrievers showing increased propensity for collapse and loss of clot engagement under tortuous conditions (16).

Similarly, in severely tortuous anatomy, direct aspiration catheters may lose stable contact with the proximal face of the thrombus, leading to incomplete aspiration and clot displacement. The higher new territory embolism rate we observed is consistent with these experimental findings and with the clinical observations of Kaesmacher et al. regarding mechanisms of reperfusion failure in stent retriever-based thrombectomy (17).

The higher rate of IA-tPA use in the tortuous group (21.0% vs. 11.4%; *p* = 0.007) likely reflects an operator-driven escalation strategy in technically challenging cases. In tortuous anatomy, difficulties in achieving effective mechanical recanalization may prompt the use of adjunctive pharmacological therapy as a salvage approach. Although IA-tPA is known to carry an inherent hemorrhagic risk, our sensitivity analysis demonstrated that the association between tortuosity and hemorrhage persisted even after excluding IA-tPA-treated patients. This suggests that while IA-tPA may contribute to bleeding risk, it does not fully account for the increased hemorrhage rates observed in tortuous patients. Instead, the interplay between anatomical complexity, procedural manipulation, and selective use of adjunctive therapies likely represents a multifactorial mechanism underlying hemorrhagic complications in this subgroup. This finding may suggest a more frequent need for pharmacological rescue in the setting of incomplete mechanical recanalization in anatomically complex cases.

The substantially higher prevalence of prior stroke in the tortuous group (22.9% vs. 9.5%; *p* < 0.001) merits further interpretation. Vascular tortuosity of the cerebral arteries has been associated with hemodynamic abnormalities—specifically, increased wall shear stress and turbulent flow at angulations—that promote endothelial dysfunction, platelet activation, and accelerated atherogenesis (18, 19).

The higher prior stroke prevalence in the tortuous group may reflect a chronically pro-thrombotic vascular milieu associated with siphon tortuosity, rather than simply a chance association. To address potential confounding, we performed a sensitivity analysis excluding patients with CAD and prior CABG. The results remained consistent, with no significant difference in first-pass success between tortuous and non-tortuous groups (*p* = 0.616). Importantly, the aspiration technique remained significantly associated with higher first-pass success (*p* < 0.001) and improved 90-day functional outcomes (*p* = 0.034). These findings suggest that the main results are robust and not solely driven by baseline differences in cardiovascular comorbidities.

Perhaps the most clinically actionable finding of our study is the performance of the aspiration technique in tortuous patients. A direct aspiration First Pass thrombectomy (ADAPT) approach achieved a first-pass success rate of 72.5% in the tortuous subgroup, compared to 35.9 and 38.4% with combined and isolated stent retriever approaches, respectively (*p* < 0.001). This translated into significantly shorter procedural times (median 41 vs. 53 min; *p* = 0.004) and fewer passes (median 2 vs. 4; *p* < 0.001). The predominance of 6F aspiration catheters in the primary aspiration subgroup may have contributed to the observed procedural advantage and should be considered when interpreting technique-specific outcomes. While final eTICI scores and 90-day mRS showed numerical trends favoring aspiration (75.0% mRS 0–2 vs. 57.6% combined and 54.8% isolated SR), these differences did not achieve statistical significance, possibly due to insufficient sample size within the tortuous subgroup for subgroup analyses.

The theoretical basis for aspiration superiority in tortuous anatomy is compelling. Unlike stent retrievers, which require sufficient trackability and radial force to expand and engage the clot in a straight configuration, aspiration catheters function primarily through suction mechanics, requiring a stable position adjacent to the proximal clot face rather than geometric co-axility with the vessel lumen. Sharp angulations may actually assist aspiration catheter seating by providing wall contact that prevents catheter withdrawal under suction force. Furthermore, the flexibility of modern large-bore aspiration catheters (e.g., SOFIA, Penumbra ACE) allows them to conform to tortuous anatomy while maintaining suction. These findings are consistent with the broader literature favoring aspiration in complex anatomies, as reviewed by Kaneko et al. (15). Regarding the technical conduct of ADAPT in tortuous cavernous ICA anatomy, aspiration catheters were advanced using coaxial microcatheter/microwire support when catheter tracking was difficult. This was not treated as a mandatory step in every case, but rather as an operator-selected maneuver according to access stability, catheter support, and the geometry of the cavernous siphon. This clarification is relevant because severe siphon angulation may predispose to catheter kinking, loss of pushability, and difficulty in maintaining stable contact with the thrombus.

Our data provide specific quantitative support for preferential use of aspiration in the cavernous ICA tortuosity context.

Although worse early functional outcomes were observed in the tortuous group at discharge, the difference in 90-day functional outcomes was not statistically significant. This apparent convergence may reflect delayed neurological recovery, potentially driven by gradual reperfusion, collateral circulation dynamics, and the effects of post-stroke rehabilitation.

Importantly, mortality at 90 days could not be analyzed separately, as outcomes were recorded in a dichotomized format (mRS 0–2 vs. 3–6). Therefore, the potential contribution of differential mortality to this convergence cannot be excluded and represents a limitation of the present study.

However, the 6.8 percentage-point difference at 90 days, while not statistically significant, remains clinically meaningful; a larger study would be required to definitively determine whether this difference reaches significance.

Lin et al. originally applied their cavernous ICA classification to predict procedural complexity in Pipeline embolization for intracranial aneurysms, demonstrating that Types III and IV were associated with significantly longer procedure times and greater technical difficulty (6).

Our study is, to our knowledge, the first to apply this validated classification system specifically to the MT context for AIS, providing evidence that the same anatomical categories predict meaningful differences in MT efficiency, complication rates, and early functional outcomes. This supports the inclusion of cavernous ICA morphology assessment—readily performed on pre-procedure CTA or at the time of initial diagnostic angiography—as a standard component of MT planning.

Several limitations of the present study warrant acknowledgment. First, the retrospective single-center design introduces potential selection and operator bias, and the findings should be interpreted within the context of a high-volume center with experienced neurointerventionalists; generalizability to lower-volume centers may differ. Second, technique selection was not randomized, precluding causal inference regarding the aspiration technique advantage in the tortuous subgroup. One important limitation of this study is the lack of detailed clot-related imaging parameters. Specifically, quantitative assessment of thrombus characteristics such as clot density (Hounsfield Units), clot length, and the presence of hyperdense artery sign were not systematically recorded. As a result, potential differences in clot composition between treatment groups could not be evaluated. This is particularly relevant, as operators may have preferentially selected specific thrombectomy techniques based on perceived clot characteristics in real-world practice. Third, patients with ICA occlusion and tandem occlusions were excluded, which limits generalizability to these technically challenging subgroups. Fourth, the sample size within the tortuous subgroup technique comparisons (particularly the aspiration group, *n* = 40) may have been insufficient to detect significant differences in secondary outcomes such as final eTICI and 90-day mRS. Therefore, the lack of statistical significance in these outcomes should be interpreted with caution and should not be considered as evidence of equivalence between techniques. Prospective, randomized studies or large multicenter registries stratifying technique by vascular anatomy will be necessary to definitively establish optimal technique selection in tortuous cavernous ICA patients. Although the present study focused specifically on cavernous ICA tortuosity, extracranial carotid artery tortuosity may also influence catheter navigation, puncture-to-first-pass time, and overall technical complexity during mechanical thrombectomy, as recently reported by Lovoković et al. (20). Therefore, the lack of systematic assessment of extracranial ICA tortuosity represents an additional limitation of this study.

## 5 Conclusion

Cavernous ICA tortuosity of Lin types III–IV is an independent anatomical predictor of reduced first-pass recanalization success and is associated with a higher procedural complexity burden, increased intracranial hemorrhage risk, more frequent new territory embolism, and inferior short-term functional outcomes in patients undergoing mechanical thrombectomy for MCA occlusion acute ischemic stroke. Among patients with tortuous cavernous ICA anatomy, the aspiration technique was associated with markedly superior first-pass success, shorter procedural times, and fewer device passes compared to combined or isolated stent retriever approaches, without an increase in complication rates.

These findings have direct clinical implications: pre-procedural identification of cavernous ICA tortuosity on CTA or diagnostic angiography should be incorporated into routine MT planning, and aspiration as the primary thrombectomy strategy should be considered preferentially in patients with Lin type III–IV cavernous ICA morphology. Validation through prospective multicenter studies and, ultimately, randomized controlled trials comparing technique selection strategies in anatomically defined patient subgroups is warranted.

## Data Availability

The data supporting the findings of this study are included in the article and Supplementary material. Additional anonymized data may be made available from the corresponding author upon reasonable request, subject to institutional and ethical restrictions.
