# Supplementary material for: Impact of cavernous internal carotid artery tortuosity on technical and clinical outcomes in mechanical thrombectomy for acute ischemic stroke: a single-center retrospective study
Source: Front Neurol. 2026 Aug 5;17:1867423. doi: 10.3389/fneur.2026.1867423 (PMC13488311; doi:10.3389/fneur.2026.1867423)
Supplement: Supplementary file 1 [file Supplementary_file_1.DOCX]

A

B

C

D

E

F

G

H

Supplementary Figure S1. Representative angiographic images of a patient with cavernous internal carotid artery (ICA) tortuosity. Based on the Lin classification system, the cavernous ICA was classified as Type IV tortuosity. (A) Left cervical ICA angiography. (B–E) Pre-procedural angiographic images demonstrating severe tortuosity of the cavernous ICA segment. (F–H) Final angiographic runs following mechanical thrombectomy, demonstrating restoration of intracranial arterial flow.
