# Supplementary material for: Impact of cavernous internal carotid artery tortuosity on technical and clinical outcomes in mechanical thrombectomy for acute ischemic stroke: a single-center retrospective study
Source: Front Neurol. 2026 Aug 5;17:1867423. doi: 10.3389/fneur.2026.1867423 (PMC13488311; doi:10.3389/fneur.2026.1867423)
Supplement: Supplementary file 2 [file Table_1.DOCX]

.In the sensitivity analysis excluding patients with coronary artery disease and prior CABG, no significant difference in first-pass success was observed between tortuous and non-tortuous groups (p=0.616), while the aspiration technique remained significantly associated with both higher first-pass success and improved 90-day functional outcomes. (supp. Table-1)

**Supplementary Table 1. Sensitivity analysis excluding patients with coronary artery disease and prior coronary artery bypass grafting**

| **Variable** | **Group** | **n (%)** | **p value** |
| --- | --- | --- | --- |
| **First-pass recanalization** | ADAPT | 42 (70.0%) | **<0.001** |
|  | Combined | 91 (38.4%) |  |
| **90 day mRS (0–2)** | ADAPT | 46 (76.7%) | **0.034** |
|  | Combined | 147 (62.0%) |  |
| **First-pass recanalization** | Non-tortuous | 59 (46.5%) | 0.616 |
|  | Tortuous | 74 (43.5%) |  |

Data are presented as number (percentage). CAD: coronary artery disease; CABG: coronary artery bypass grafting. Analysis was performed after excluding patients with CAD and prior CABG

In the sensitivity analysis excluding IA-tPA-treated patients, tortuous cavernous ICA anatomy remained significantly associated with higher hemorrhage rates (35.2% vs. 22.1%, p=0.006). In contrast, the association between thrombectomy technique and hemorrhage was no longer statistically significant (p=0.073). (supp. Table-2)

**Supplemenary Table 2. Sensitivity analysis excluding IA-tPA patients**

| **Cavernous tortuosity** | **No hemorrhage n (%)** | **Hemorrhage n (%)** | **Total** | **p-value** |
| --- | --- | --- | --- | --- |
| Non-tortuous | 152 (77.9%) | 43 (22.1%) | 195 | **0.006** |
| Tortuous | 105 (64.8%) | 57 (35.2%) | 162 |  |
| **Technique** |  |  |  |  |
| Combined | 111 (66.5%) | 56 (33.5%) | 167 | 0.073 |
| Aspiration | 54 (74.0%) | 19 (26.0%) | 73 |  |
| Stent retriever alone | 92 (78.6%) | 25 (21.4%) | 117 |  |

Data are presented as number (row percentage). IA-tPA: intra-arterial tissue plasminogen activator. A sensitivity analysis was performed after excluding patients treated with IA-tPA.
